# Genetic Screening Revealed the Negative Regulation of miR-310~313 Cluster Members on Imd Pathway during Gram-Negative Bacterial Infection in *Drosophila*

**DOI:** 10.3390/genes15050601

**Published:** 2024-05-08

**Authors:** Yao Li, Yixuan Sun, Ruimin Li, Hongjian Zhou, Shengjie Li, Ping Jin

**Affiliations:** 1 College of Plant Protection, Yangzhou University, Yangzhou 225009, China; 2 Laboratory for Comparative Genomics and Bioinformatics & Jiangsu Key Laboratory for Biodiversity and Biotechnology, College of Life Science, Nanjing Normal University, Nanjing 210046, China; liruimin920130@163.com (R.L.); zhouhongjian8189@163.com (H.Z.); 3 School of Food Science, Nanjing Xiaozhuang University, Nanjing 211171, China; lishengjie@njxzc.edu.cn

**Keywords:** miR-310-313 cluster, *Imd*, Imd pathway, innate immunity

## Abstract

Innate immune response is the first line of host defense against pathogenic microorganisms, and its excessive or insufficient activation is detrimental to the organism. Many individual microRNAs (miRNAs) have emerged as crucial post-transcriptional regulators of immune homeostasis in *Drosophila melanogaster*. However, the synergistical regulation of miRNAs located within a cluster on the Imd-immune pathway remains obscured. In our study, a genetic screening with 52 transgenic UAS-miRNAs was performed to identify ten miRNAs or miRNA clusters, including the miR310~313 cluster, which may function on Imd-dependent immune responses. The miRNA RT-qPCR analysis showed that the expression of miR-310~313 cluster members exhibited an increase at 6–12 h post *E. coli* infection. Furthermore, the overexpression of the miR-310~313 cluster impaired the *Drosophila* survival. And the overexpression of miR-310/311/312 reduced *Dpt* expression, an indication of Imd pathway induced by Gram-negative bacteria. Conversely, the knockdown of miR-310/311/312 led to increases in *Dpt* expression. The Luciferase reporter expression assays and RT-qPCR analysis confirmed that miR-310~313 cluster members directly co-targeted and inhibited *Imd* transcription. These findings reveal that the members of the miR-310~313 cluster synergistically inhibit Imd-dependent immune responses by co-targeting the Imd gene in *Drosophila*.

## 1. Introduction

Innate immunity serves as the primary defense mechanism of the host against pathogenic microorganisms. Due to its highly conserved innate immune pathways and lack of an adaptive immune system, *D. melanogaster* is an attractive model organism for studying innate immunity [1]. Similar to mammals, *Drosophila* evolves two evolutionarily conserved Nuclear Factor kappa B (NF-κB) pathways, namely, the Toll and immune deficiency (IMD) pathways, which are activated in response to pathogenic invasions [2]. The Toll pathway is primarily employed by *Drosophila* to defend against Gram-positive bacteria or fungi, while the Imd pathway is activated for combating Gram-negative bacteria [3]. The activation of the Imd pathway leads to the formation of an intracellular complex involving the Imd protein, the adaptor protein dFadd, and the caspase Dredd [4]. Subsequently, the complex recruits TGF-β activated kinase 1 (Tak1) and Tak1-associated binding protein 2 (Tab2) [5]. Tak1 phosphorylates and activates the IKK kinase complex composed of the proteins Ird5 and Kenny [6]. The active IKK complex then triggers the activation of the NF-kB transcription factor Relish (Rel) through phosphorylation [7]. Additionally, the caspase Dredd within the IKK complex cleaves the Rel into the Rel-49 and Rel68 subunits [8]. The cleaved Rel-68 translocates into the nucleus and promotes the transcription of antimicrobial peptides (AMPs) in response to pathogenic invasions [9]. Among these AMPs, the secretion of Diptericin (Dpt) is mainly regulated by the Imd pathway [10]. Currently, the activation mechanisms of Imd-dependent innate immune response have been extensively elucidated, but the regulatory mechanisms governing the Imd pathway remain insufficiently investigated.

Both the overactivation and the suppression of innate immune response can be detrimental to organisms, and even threaten survival. The proper regulation of innate immune response is indispensable for defending against pathogens and protecting the host’s tissues [11]. So far, various activators and inhibitors of the Imd pathway have been identified at multiple regulatory levels in *Drosophila*. For instance, STING, sick, Akirin, and E3-ligase inhibitor of apoptosis 2 (Iap2) positively regulate the Imd pathway via the activation of Rel and Dredd expression [12,13]. On the contrary, some inhibitors can negatively regulate the Imd pathway to maintain innate immune homeostasis, such as PGRP-LF, pirk, dUSP36, Trabid, CYLD, Caspar, and Dnr1 [13,14]. MicroRNAs (miRNAs), as a class of small non-coding RNA molecules, were also found to fine-tune *Drosophila* Imd-dependent immune responses. Only *Drosophila* miR-34 functions as a positive regulator of the Imd pathway by targeting *Dlg1* and *Eip75B* [15]. Conversely, seven miRNAs have been reported as negative regulators of the Imd pathway [16,17,18,19]. Notably, miR-9a, miR-981, and let7 target the same gene, *Dpt*, to suppress Imd-dependent immune responses [17,20]. The co-targeting of multiple miRNAs to the same gene effectively enhances the fine-tuning regulation of an individual miRNA. Although most clustered miRNAs share similar seed sequences, it remains a mystery whether the members of these miRNA clusters work together synergistically in regulating the Imd pathway.

The miR-310~313 cluster is located on chromosome 2R of *Drosophila* and consists of four members, namely, miR-310, miR-311, miR-312, and miR-313, which exhibit a conserved sequence. Multiple studies have demonstrated the regulatory role of the miR-310 cluster in *Drosophila* development, insecticide resistance, and innate immunity. The miR-310 cluster members regulated cell signaling and cell adhesion in early somatic and germline progenitor cells by targeting Arm, thereby facilitating normal differentiation in the *Drosophila* male testis [21]. Another two studies showed that miR-310~313 cluster members exert an influence on brain development and the sculpting of haltere morphologies via suppressing the muscular dystrophy-associated extracellular matrix receptor dystroglycan and Ubx fine-grain expression in *Drosophila* [22,23]. MiR-310 cluster members also enhanced the insecticide susceptibility of DDT-resistant 91-R flies via targeting P450 detoxification genes, *Cyp6g1* and *Cyp6g2* [24]. Our previous work has confirmed that these miR-310~313 cluster members suppressed the activity of the Toll pathway by targeting the 3′UTR of *Drosomycin* (*Drs*), an AMP produced by Toll signaling [25]. In this study, we firstly employed the transgenic UAS-miRNA library to screen for the potential role of the miR-310~313 cluster in Imd-dependent immune responses. Secondly, the expression patterns of miR-310~313 cluster members were detected at different time-points post Gram-negative bacterial infection. Thirdly, the effect of miR-310~313 cluster members on the activity of the Imd pathway and survival induced by Gram-negative bacteria was examined. Lastly, the co-targeting mechanism of miR-310~313 cluster members to the Imd pathway was revealed in vitro and in vivo. By integrating the previously observed inhibitory effect of the miR310~313 cluster on the Toll pathway, these findings unveil a dual regulatory role of the miR310~313 cluster in *Drosophila* innate immune response against Gram-negative and Gram-positive bacterial infection.

## 2. Materials and Methods

### 2.1. Fly Stocks and Genetics

Fly stocks were maintained and crossed at 25 °C on standard medium, while the stocks with tub-G80^ts^ were cultured at 18 °C according to experimental requirements. The following fly stocks were obtained from the FlyORF (Zurich, Switzerland) or Bloomington Drosophila Stock Center (Bloomington, IN, USA), including wild-type (W^1118^), tubulin-Gal4/TM3,sb,ser (tub-Gal4/+), daughter-Gal4 (da-Gal4), tubulin-Gal80^ts^;TM2/TM6B (tub-G80^ts^), UAS-miR-310, UAS-miR-310 sponges (UAS-miR-310 SP), UAS-miR-311, UAS-miR- 311 sponges (UAS-miR-311 SP), UAS-miR-312, UAS-miR-312 sponges (UAS-miR-312 SP), UAS-miR-313, UAS-miR-313 sponges (UAS-miR-313 SP), and other 52 UAS-miRNA lines. The *Drosophila* line tubulin-Gal80^ts^;tubulin-Gal4/TM6B (tub-G80^ts^;tub-Gal4/+) was generated through the crossing of tub-G80^ts^, da-Gla4, and tub-Gal4/+ as mentioned in our previous study [25].

### 2.2. Infection and Genetic Screening

Adult male flies from control, treatment with ectopically expressed miRNAs, and treatment with miRNAs knockdown were collected at 3–4 days after eclosion and subsequently infected with *Escherichia coli*. These infection experiments were performed using a Nanoject apparatus (Nanoliter 2010, WPI, Sarasota, FL, USA) by injecting 10 nL microbial suspensions (PBS as injury control) into the thorax of each adult. Infected flies were collected at specific time points for subsequent experimental analysis.

To avoid the potential lethality or developmental abnormalities associated with miRNA overexpression in *Drosophila*, a screening platform was established for the diverse assessment of miRNA gain-of-function in innate immunity using 52 transgenic UAS-miRNA lines and temperature-sensitive Gal4 drivers (tub-G80^ts^; tub-G4/+). Firstly, each of the 52 UAS-DsRde-pre-miRNA lines was subjected to crosses with tub-G4 and da-G4. Secondly, a temperature-sensitive tub-G80^ts^; tub-G4/+ line was introduced into miRNA overexpression lines (tub-Gal80^ts^; tub-Gal4 > miRNAs) to achieve temperature-induced miRNA overexpression in vivo [26]. Thirdly, progenies generated from the above cross were initially raised at 18 °C permissive temperature, which effectively blocked Gal4 > miRNA expression through the activation of Gal80^ts^ expression. After adult emergence, the progenies were transferred to a temperature-controlled environment at 29 °C, which limited Gal80^ts^ expression and released Gal4 protein to allow miRNA expression. Upon infection of *E. coli*, the expression level of AMP *Dpt*, as an indication Imd-immune activation, was checked in these miRNA overexpression lines using RT-qPCR. The schematic diagrams illustrating the Gal80^ts^/Gal4-UAS binary system were drawn use BioRender, an online graphic design software (https://www.biorender.com, accessed on 25 April 2024).

### 2.3. Survival Experiments

Post-infection survival is the most comprehensive and common method to assess immune response deficiencies [27]. The tub-Gal80^ts^; tub-Gla4/+ and tub-Gal80^ts^; tub-Gal4 > miRNAs were treated using PBS and *Enterobacter cloacae*, as a Gram-negative bacterium that can induce Imd-immune death in *Drosophila*. The survival rate of these flies was monitored every 6 h (h) for 5 days (d) after infection with *E. cloacae*. There were ≥100 flies/group.

### 2.4. Real-Time Quantitative Polymerase Chain Reaction (RT-qPCR)

TRIzol reagent (Invitrogen, Carlsbad, CA, USA) was used to isolate Total RNAs from wild-type (W^1118^) flies, control flies (tub-Gal80^ts^; tub-Gla4/+) and tub-Gal80^ts^; tub-Gal4 > miRNAs injected by PBS or *E. coli* at 3 h, 6 h, 12 h, 24 h, and 48 h following the instructions. The quality of total RNA was evaluated using spectrophotometry (NanoDrop, Thermo Scientific, Waltham, MA, USA), with a concentration of 500 ng and a purity OD260/230 ratio ranging from 1.8 to 2.2. The cDNA for miRNA RT-qPCR and gene RT-qPCR was synthesized using a miRNA 1st Strand cDNA Synthesis kit (by stem-loop) (Vazyme, Nanjing, China) and a 1st strand cDNA synthesis kit (Vazyme, Nanjing, China) under the following reaction conditions: initial denaturation at 25 °C for 5 min, followed by incubation at 50 °C for 15 min, and final extension at 85 °C for 5 min. The AceQ SYBR Green Master Mix (Vazyme, Nanjing, China) was utilized for miRNA RT-qPCR and gene RT-qPCR on ABI Step One fast real-time PCR instrument (Applied Biosystems, Foster, CA, USA) as follows: denaturation for 3 min at 95 °C, followed by 40 cycles at 95 °C for 10 s, and 60 °C for 30 s [28]. The CQ value of mRNA and miRNA transcript were normalized to rp49 and 5 sRNA, respectively. The gene or miRNA expression assays were performed with three technical replicates and three biological replicates for each treatment. Data analysis was conducted using the relative 2^−△△CT^ method.

### 2.5. Bioinformatics Analysis

The mature sequences of miR-310/311/312/313 in *Drosophila* were obtained from miRbase (https://www.mirbase.org/hairpin/MI0000423, accessed on 20 May 2017). The targets of miR-310~313 were predicted using TargetScan (www.targetscan.org/fly_12/, release 7.2 October 2018, accessed on 20 May 2017) [29,30].

### 2.6. Luciferase Reporter Expression Assays

Based on *Drosophila* genomics, we obtained and amplified the hairpin sequences of miR-310, miR-311, miR-312, and miR-313, and the CDS+3′UTR sequence of *Imd* using RT-PCR. All DNA sequences of the four miRNAs and Imd CDs+3′UTR were recombined into pAc5.1/V5-HizA vector (Invitrogen, Carlsbad, CA, USA) to construct miRNA expression plasmids (pAc-miR-310, pAc-miR-311, pAc-miR-312, and pAc-miR-313) and target reporter expression plasmid (pAc-luc Imd CDS+3′UTR), respectively. The remaining plasmids used in this study were procured from Promega company. Then, the miRNA and target reporter gene expression vector were co-transfected into *Drosophila* S2 cells, followed by the evaluation of luciferase activity at 48 h post-transfection. Dual Luciferase Reporter Assay System (Promega, Madison, WI, USA) was used to detect luciferase activity. The firefly luciferase activity was normalized as Renilla luciferase activity per transfection well. Three technical and biological replicates were conducted for each treatment.

### 2.7. Statistical Analysis

The statistical analyses utilized in this study are presented as means ± SEM. The significant differences between the two treatments were detected using a two-tailed Student’s *t*-test. For the analysis of the survival experiment, the log-rank (Mantel–Cox) test was employed. GraphPad Prism 10.2 was used for final plotting and data analysis.

## 3. Results

### 3.1. Genetic Screening of miRNAs Regulating Drosophila Imd Pathway

**Figure 1:**
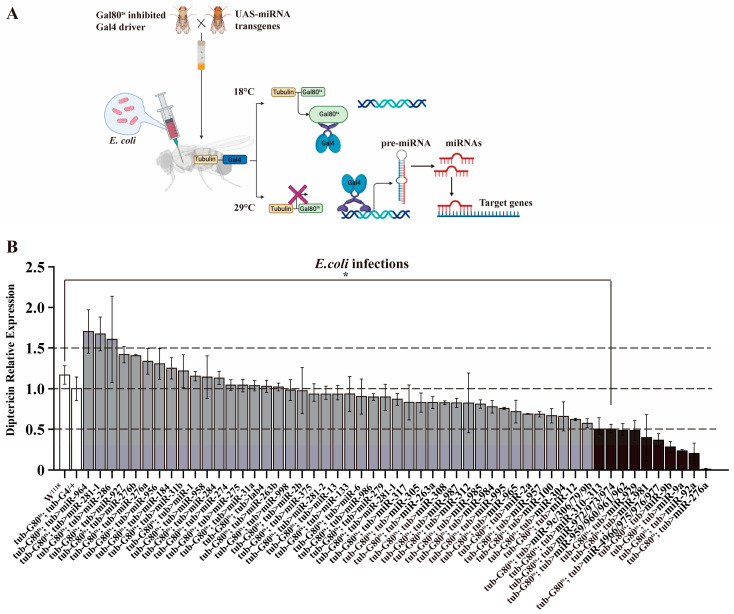
Genetic screening of Imd pathway-relative miRNAs. (**A**) Schematic diagram illustrating the Gal80^ts^/Gal4-UAS binary system for systematic in vivo screens of activated miRNA or miRNA clusters. (**B**) The relative expression level of *Dpt* in ectopic miRNA transgenes with *E. coli* infection. The differential expressions of *Dpt* with a two-fold change are shown with blank columns. The *Dpt* expression levels of control (tub-Gal80^ts^; tub-Gal4/+) and WT (W^1118^) flies are shown with black columns. Dotted lines represent ± two-fold changes (*n* = 3, error bars represent standard error). *t*-test, * *p* < 0.05.

Based on the previously small RNA seq-identified miRNAs in *Drosophila* infected with *E. coli* [20], a set of 52 temperature-sensitive miRNA overexpression lines (tub-Gal80^ts^; tub-Gal4 > miRNAs) were generated for genetic screening. In these miRNA overexpression lines, Gal80^ts^ inhibits the activity of Gal4 and prevents miRNA expression at the permissive temperature of 18 °C, but not at the restrictive temperature of 29 °C (Figure 1A). Using this system, we detected the relative expression levels of *Dpt* in miRNA-overexpressing flies infected with *E. coli*, which can serve as an indicative measure of miRNAs involved in Imd signaling. A total of ten miRNAs or miRNA clusters were identified to reduce *Dpt* expression by more than half in flies infected with *E. coli*, including the miR-310~313 cluster, the miR972~974 cluster, the miR-959~962 cluster, miR-929, miR-981, miR-4966/975~977, miR-9b, miR-9a, miR-92a, and miR-276a (Figure 1B). The ten miRNAs or miRNA clusters, including the miR310~313 cluster, were implicated to function on Imd-dependent immune responses against Gram-negative bacterial infection.

### 3.2. The Expression of miR-310~313 Cluster Members Was Upregulated in Response to Gram-Negative Bacterial Infection

**Figure 2:**
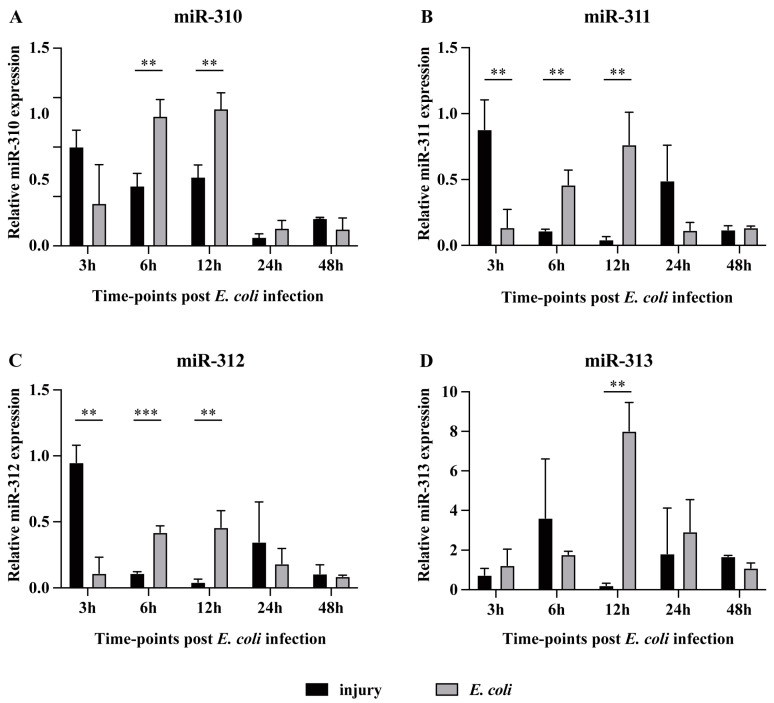
The expression levels of miR-310~313 cluster members during *E. coli* infection. The expression of miR-310 (**A**), miR-311 (**B**), miR-312 (**C**), and miR-313 (**D**) was detected in flies treated with either PBS injury or *E. coli* infection at five time-points post challenge. All RT-qPCR data are presented as the mean + SEM (*n* > 3). *t*-test ** *p* < 0.01; *** *p* < 0.001.

Among these ten miRNAs or miRNA clusters identified in the genetic screening, the overexpression of the miR-310~313 cluster had a significant effect on *Dpt* expression induced by *E. coli* (Figure 1B). To further investigate the response of miR-310~313 cluster members to *E. coli* infection, we examined the temporal expression patterns of miR-310/miR-311/miR-312/miR-313 in wide-type flies infected with *E. coli*. The expression levels of miR-310/miR-311/miR-312/miR-313 were significantly upregulated in *E. coli*-infected flies, ranging from 2.7- to 35.9-fold higher compared to the injured flies at 6 h and 12 h post challenge (Figure 2A–D). Interesting, a downregulation of the expression of miR-311/miR-312 was observed in *E. coli*-infected flies at 3 h post challenge (Figure 2B,C). This suggested that *E. coli* infection downregulated the expression levels of miR-311 and miR-312 during the initial stage, but subsequently induced the upregulation of miR-310~313 members during a robust immune response, indicating the crucial role of the miR-310~313 cluster in regulating Imd-dependent innate immune response against Gram-negative bacterial infection.

### 3.3. miR-310~313 Cluster Members Negatively Regulated Imd Pathway and Survival of Drosophila Infected with Gram-Negative Bacteria

**Figure 3:**
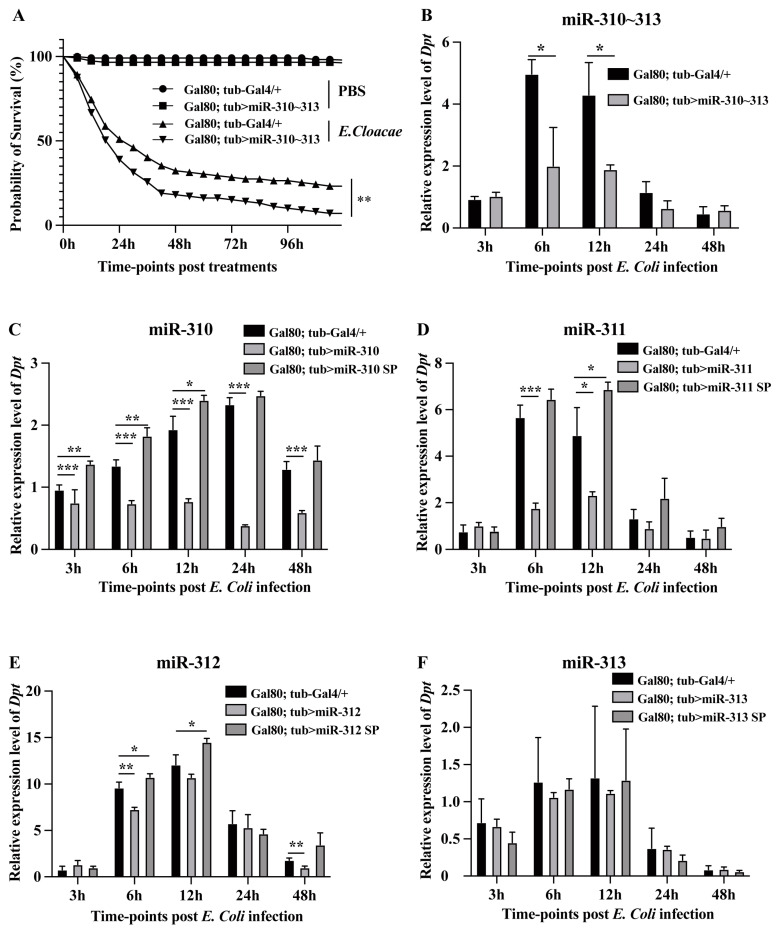
The negative regulation of miR-310~313 cluster members on Imd-dependent immune responses. (**A**) The survival rates of the miR-310~313 cluster-overexpressing flies, and the control flies were assessed at 6-hourly intervals up to 120 h after injection with PBS or *E. cloacae*, respectively. Gal80^ts^; Tub-Gal4/+ (*n* = 119) and Gal80^ts^; Tub > miR-310~313 cluster (*n* = 117) after PBS injection, Gal80^ts^; Tub-Gal4/+ (*n* = 102) and Gal80^ts^; Tub > miR-310~313 cluster (*n* = 105) after *E. cloacae* infection. The *Dpt* expression levels in (**B**) miR-310~313 cluster-overexpressing lines, (**C**) miR-310-overexpressing lines and knockdown lines, (**D**) miR-311-overexpressing lines and knockdown lines, (**E**) miR-312-overexpressing lines and knockdown lines, and (**F**) miR-313-overexpressing lines and knockdown lines were detected at 3 h, 6 h, 12 h, 24 h, and 48 h post *E. coli* and PBS injection using RT-qPCR analysis. The RT-qPCR data are presented as the mean + SEM (*n* > 3). *t*-test, * *p* < 0.05; ** *p* < 0.01; *** *p* < 0.001.

In order to evaluate the physiological role of miR-310~313 in immune response, the changes in survival rate were examined in miR-310~313 cluster-overexpressing flies upon infected with the lethal Gram-negative bacteria *E. cloacae*. The survival rate of miR-310~313 cluster-overexpressing flies exhibited a statistically significant reduction compared with that of control flies (Figure 3A). To further validate the effect of the miR-310~313 cluster on the Imd pathway, the relative expression level of *Dpt*, as the readout of Imd pathway activation, was monitored in miR-310~313-overexpressing flies at five time-points post *E. coli* infection. The overexpression of the miR-310~313 cluster in flies resulted in a significant reduction in *Dpt* expression at 6 h (59.7%) and 12 h (55.9%) post challenge, as compared to the control (Figure 3B). Consistently, significant decreases in *Dpt* expression were also observed in miR-310-, miR-311-, and miR-312-overexpressing flies after the challenge (Figure 3C–E, respectively), whereas the knockdown of miR-310/miR-311/miR-312 led to significantly increases in *Dpt* expression in miRNA sponge lines (tub-G80^ts^; tub > miR-310 SP, tub-G80^ts^; tub > miR-311 SP, and tub-G80^ts^; tub > miR-312 SP), compared with control flies at 6 h and 12 h post infection (Figure 3C–E). The results suggested that the members of the miR-310~313 cluster, except for miR-313 (Figure 3F), synergistically inhibit the Imd pathway, and, in particular, the ectopic expression of the miR-310~313 cluster disrupts the immune homeostasis of the *Drosophila* Imd pathway.

### 3.4. miR-310~313 Cluster Members Co-Targeted Imd Gene of Imd Pathway

**Figure 4:**
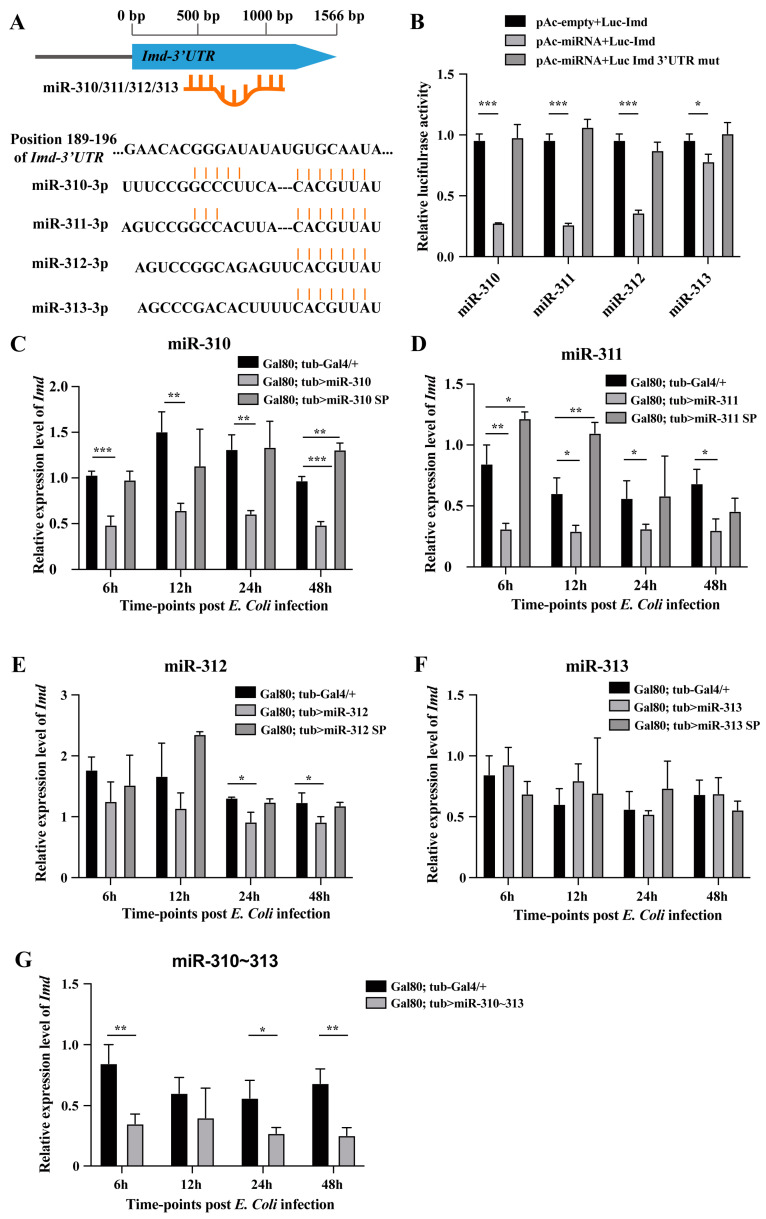
Identification of *Imd* gene directly targeted by miR-310~313 cluster members. (**A**) The target gene, *Imd*, and its potential binding site of miR-310~313 cluster members were predicted by TargetScan. (**B**) The activity of luciferase reporter containing *Imd* 3′UTR or *Imd* 3′UTR mutation was detected in S2 cells with or without miR-310~313 members via a dual luciferase reporter assay. The *Imd* expression levels in (**C**) miR-310-overexpressing lines and knockdown lines, (**D**) miR-311-overexpressing lines and knockdown lines, (**E**) miR-312-overexpressing lines and knockdown lines, (**F**) miR-313-overexpressing lines and knockdown lines, and (**G**) miR-310~313 cluster-overexpressing lines were detected at 6 h, 12 h, 24 h, and 48 h post *E. coli* and PBS injection using RT-qPCR analysis. The RT-qPCR data are presented as the mean + SEM (*n* > 3). *t*-test, * *p* < 0.05; ** *p* < 0.01; *** *p* < 0.001.

To further elucidate the inhibitory mechanism of miR-310~313 cluster members on the Imd pathway, TargetScan algorithms was used to predict the putative target genes of miR-310, miR-311, miR-312, and miR-313. Our results showed that miR-310~313 cluster members have potential to bind the 3′UTR of the *Imd* transcript, which is the crucial protein in recognizing Gram-negative bacteria and initiating the Imd pathway (Figure 4A). The luciferase reporter assay in vitro confirmed that miR-310~313 cluster members exhibited significantly reductions of 62.3–72.6% in the activity of luciferase reporter harboring the 3′UTR region of the *Imd* transcript (Figure 4B). Moreover, the introduction of a target site mutation in the *Imd* 3′UTR restored the reporter activity to normal levels (Figure 4B). In vivo the expression levels of *Imd* in miR-310-, miR-311-, miR-312-, and miR-310~313 cluster-overexpressing flies were significantly lower than the control flies with *E. coli* infection during Imd-dependent immune responses (Figure 4C–E,G). In addition, the expression levels of *Imd* were restored or significantly upregulated in miR-310, miR-311, and miR-312 knockdown flies compared to the control flies (Figure 4C–E,G). Our finding indicated that miR-310~313 members exert an inhibitory effect on Imd-dependent immune responses via directly co-targeting the *Imd* gene.

## 4. Discussion

MiRNAs predominantly function as regulators in innate immunity by exerting post-transcriptional repression on immune-related gene expression [31,32]. Increasing evidence has indicated that multiple miRNAs regulate distinct immune pathways in Drosophila. Six miRNAs or miRNA clusters (miR-8, miR-958, miR-317, miR-964, the miR-310~313 cluster, and the miR-959~962 cluster) were reported to regulate Toll-dependent immune response by targeting Toll, Dif, Dorsal, and Drs [25,33,34]. Seven miRNAs or miRNA clusters were validated to regulate Imd-dependent immune responses, namely, miR-34, miR-277, miR-317, miR-275, miR-9a, miR-981, and let7 [19,20,21,22,23,24]. Our genetic screening found that ten miRNAs or miRNA clusters participated in the regulation of Imd-dependent immune responses against Gram-negative bacterial infection (Figure 1B). The ten miRNAs identified in our study included well-studied ones, such as miR-9a and miR-981 [21,24]. Additionally, our genetic screening platform successfully identified eight previously unexplored miRNAs or miRNA clusters that may play a crucial role in the regulation of the Imd pathway. The function of these eight miRNAs or miRNA clusters in the Imd pathway requires further experimental verification.

In our work, the expression patterns of miR-310/311/312 exhibited synchronized fluctuations during *Drosophila* immune response against Gram-negative bacterial infection (Figure 2). Meanwhile, these three miRNAs showed a significant negative regulation of the Imd pathway (Figure 3). The activity of the Imd pathway has been widely acknowledged to occur as early as 3 h post *E. coli* infection, reaching its peak at 6–12 h [10,23]. The *E. coli*-induced downregulation of miR-310~313 members’ expression at 3 h facilitates the initial activation of the Imd pathway. Conversely, the upregulation of miR-310~313 members by *E. coli* stimulation at 6–12 h precisely inhibits the excessive activation of Imd-dependent immune responses. Unlike the other three members, miR-313 exhibited no significant inhibitory effect on the Imd gene and Imd pathway, despite its high expression at 12 h post challenge and its ability to target the 3′ UTR of *Imd* in vitro (Figure 2, Figure 3 and Figure 4). Because miR-313 is only about 5% as abundantly transcribed as the other three members of miR-310~313 in *Drosophila* [30], this may be the reason why miR-313 overexpression or knockdown alone has no obvious inhibitory effect on the Imd pathway.

Although several studies have elucidated the regulatory mechanism of single miRNAs in *Drosophila* innate immune responses, there are limited reports on the co-regulation of miRNA cluster members in innate immune response. To date, only our two studies focused on the regulation of clustered miRNAs in *Drosophila* immunity. miR-959-962 cluster members exhibited distinct target specificities towards Toll-related genes, such as *tube*, *dorsal*, and *Toll*, thereby weakening the immune response against Gram-positive bacterial infection [34]. Moreover, miR-310~313 cluster members co-targeted *Drs* to negatively regulate Toll-immune response [25]. In this work, miR-310~313 cluster members were also found to co-target Imd, thereby performing a negative regulation on Imd-immune response (Figure 3 and Figure 4). The *Drosophila* miRNA-310~313 cluster underwent duplication and evolutionary divergence from the ancestral miRNA genes (miR-92a/b), resulting in the acquisition of similar seed sequences among its four members [35]. Hence, our findings demonstrated that miR-310~313 cluster members exhibited a preferential targeting of the same gene to suppress innate immune responses, and possessed a dual regulatory mechanism for simultaneously modulating both Toll and Imd pathways.

## 5. Conclusions

This study established an efficient genetic screening platform to identify miRNAs that regulate Imd-dependent immune responses in *Drosophila*. Based on the screening results, our further findings demonstrate that Gram-negative bacteria infection leads to a increase in the expression of miR-310~313 cluster members. And the increasing expression level of miR-310~313 cluster members inhibit the excessive activation of the Imd pathway during a robust immune response. Finally, we revealed Imd gene as a co-target of miR-310~313 cluster members. These results not only lay a foundation for uncovering the regulation role of miRNAs in Imd-dependent immune responses, but also reveal the synergistic inhibitory effect of clustered miRNAs on innate immunity.

## Data Availability

The original contributions presented in the study are included in the article. Further inquiries can be directed to the corresponding authors.
